# Video Versus Direct Laryngoscopy for Tracheal Intubation of Critically Ill Adults: A Systematic Review and Meta-Analysis

**DOI:** 10.3390/jcm14061933

**Published:** 2025-03-13

**Authors:** Paola P. Polo, Rodrigo Ramirez-Rodriguez, Rodrigo Alejandro-Salinas, Judith Yangali-Vicente, Oriana Rivera-Lozada, Joshuan J. Barboza

**Affiliations:** 1 Departamento de Anestesia, Hospital Serena del Mar, Cartagena de Indias 130001, Colombia; paola.polo@chsm.com; 2 Instituto Politécnico Nacional, 09620 Ciudad de México, Mexico; rodriguez.neuroscience@gmail.com; 3 Escuela de Medicina, Universidad Peruana de Ciencias Aplicadas, Lima 15023, Peru; roalejandro99@gmail.com; 4 Dirección General de Investigación, Universidad Inca Garcilaso de la Vega, Lima 15084, Peru; judithsyv@gmail.com; 5 Vicerrectorado de Investigación, Universidad Señor de Sipan, Chiclayo 14002, Peru; riveraoriana@uss.edu.pe; 6 Escuela de Medicina, Universidad Señor de Sipan, Chiclayo 14002, Peru

**Keywords:** video laryngoscopy, direct laryngoscopy, airway management, critically ill, systematic review, meta-analysis

## Abstract

**Background/Objectives:** Endotracheal intubation in critically ill patients presents significant challenges due to anatomical and physiological complexities, making airway management crucial. Video laryngoscopy (VL) has emerged as a promising alternative to direct laryngoscopy (DL), offering improved and higher success rates. This systematic review and meta-analysis evaluated the comparative efficacy and safety of VL versus DL in critically ill adults. **Methods:** A systematic search was conducted in PubMed, Embase, and Cochrane Library through August 2024 following PRISMA-2020 guidelines. Randomized controlled trials comparing VL and DL in critically ill adult patients were included. The RoB 2.0 tool assessed bias, and GRADE evaluated the certainty of evidence. The primary outcome was first-attempt success; secondary outcomes included intubation time, glottic visualization, and complications. Random effects models were used for data synthesis. **Results:** Fifteen studies (4582 intubations) were included. VL improved first-attempt success rates (RR 1.12; 95% CI: 1.04–1.21; I^2^ = 87%). It also reduced esophageal intubation (RR 0.44; 95% CI: 0.26–0.75), dental injuries (RR 0.32; 95% CI: 0.16–0.67), and poor glottic visualization. No significant differences were found in hypoxemia, hypotension, or mortality. **Conclusions:** VL enhances intubation success and reduces specific complications, particularly in difficult airways. However, high heterogeneity and low certainty of evidence warrant further studies to clarify its impact on critical patient outcomes.

## 1. Introduction

Emergency endotracheal intubation in critically ill patients remains a high-stakes procedure, where the choice of intubation technique can significantly impact patient outcomes. Although direct laryngoscopy has been the primary approach since the 1940s with the introduction of Miller and Macintosh laryngoscopes, it presents notable challenges in emergency settings, with success rates varying between experienced practitioners and trainees [1].

The implementation of video laryngoscopy has introduced new perspectives in airway management. Studies have demonstrated that video laryngoscopy provides superior glottic compared to direct laryngoscopy (POGO scores 88.25 ± 22.06 vs. 57.25 ± 29.26, *p* < 0.001) and potentially faster intubation times (32.90 ± 8.69 vs. 41.33 ± 15.29 s, *p* = 0.004) in controlled settings [2]. However, the translation of these advantages to emergency scenarios remains complex, with success rates influenced by multiple factors, including operator experience, patient characteristics, and clinical circumstances.

Several anatomical and clinical factors can increase intubation difficulty in emergency settings. These include limited head and neck mobility, high Mallampati scores, restricted mouth opening, and reduced thyromental distance. The impact of these factors varies significantly, with reported difficult airway rates between 6.8% for glottic exposure and 8.2% for mask ventilation in emergency departments [3]. Video laryngoscopy has emerged as a potential solution to these challenges, particularly in predicted difficult airways, where it has demonstrated a higher success rate (80.0% vs. 50.4%, *p* < 0.001) compared to direct laryngoscopy [4].

The comparative effectiveness of video versus direct laryngoscopy shows notable variation, depending on operator experience and clinical context. Recent data suggest that while video laryngoscopy may improve first-attempt success rates (93.6% vs. 80.8%, *p* < 0.001; risk difference 0.128, 95% CI: 0.0771–0.181), its utility can be influenced by specific predictors, including head and neck positioning (OR 1.63, 95% CI: 1.14–2.31), mouth opening limitations (OR 1.18, 95% CI: 1.02–1.36), and specific surgical procedures [5].

Despite these advances, significant knowledge gaps persist regarding the optimal application of video laryngoscopy in emergency settings. The interplay between device selection, operator proficiency, and patient characteristics remains incompletely understood, particularly in scenarios involving difficult airways managed by experienced clinicians. Furthermore, while video laryngoscopy has demonstrated benefits in controlled environments, its role in emergency airway management requires further clarification, especially concerning factors that might influence success rates and complications.

This systematic review and meta-analysis aimed to evaluate the comparative efficacy of video versus direct laryngoscopy for tracheal intubation in critically ill adults. By synthesizing the available evidence, we seek to provide insights into the optimal selection of laryngoscopy techniques in emergency settings, considering patient characteristics and operator experience levels.

## 2. Materials and Methods

This systematic review and meta-analysis was conducted following PRISMA-2020 guidelines and was prospectively registered in PROSPERO (CRD42024613946). The review protocol outlined the objectives, inclusion criteria, search strategy, and planned analyses to ensure methodological transparency and minimize reporting bias.

### 2.1. Searches

A comprehensive search was conducted in PubMed, Embase, Web of Science, and the Cochrane Library from inception until 8 August 2024, following PRISMA-2020 guidelines. The search strategy included a combination of MeSH terms and free-text keywords related to ‘Critically Ill Adults’, ‘Tracheal Intubation’, ‘Video Laryngoscopy’, and ‘Direct Laryngoscopy’. Boolean operators (AND/OR) were applied to refine results, and language restrictions were not imposed. The full search strategy for each database is provided in Supplementary Table S1.

Additionally, reference lists of relevant systematic reviews and included studies were manually screened for potentially eligible trials. The retrieved studies were screened in duplicate by two independent reviewers using the Rayyan platform, and discrepancies were resolved by a third investigator (JJB).

### 2.2. Eligibility Criteria

All studies that met the following criteria were included in this study: Phase 2 or Phase 3 randomized controlled trials that treated critically ill patients requiring tracheal intubation in an intensive care unit or emergency room setting, where video laryngoscopy was used to secure the airway as the intervention compared to direct laryngoscopy.

Conference abstracts, systematic reviews, narrative reviews, case reports and series, and letters to the editor were excluded.

### 2.3. Outcomes

Based on the PICO framework, the primary outcomes of this study included first-attempt intubation success (O) in critically ill adult patients (P) undergoing tracheal intubation with video laryngoscopy (I) compared to direct laryngoscopy (C). Secondary outcomes included time to successful intubation, glottic visualization, hypoxaemia, hypotension, tooth injury, esophageal intubation, and mortality.

The primary outcomes were the first-attempt success rate (defined as placement of an endotracheal tube in the trachea with a single insertion of a laryngoscope blade into the mouth and either a single insertion of an endotracheal tube into the mouth or a single insertion of a bougie into the mouth followed by a single insertion of an endotracheal tube into the mouth), time to successful intubation (defined as the interval [in seconds] between the first insertion of a laryngoscope blade into the mouth and the final placement of an endotracheal tube or tracheostomy tube in the trachea), and glottic view grade (Cormack–Lehane grade of glottis: Reflects glottis. Score range: 1 [sound] to 4 [no glottis]). The secondary outcomes were hypoxaemia (pulse arterial saturation of less than 90%), hypotension (arterial systolic pressure of less than 90 mm Hg), tooth injury, esophageal intubation, and mortality.

### 2.4. Data Extraction

After the electronic searches, the results were compiled in a single library, and duplicates were eliminated. Then, the first screening step was performed, evaluating the titles and abstracts and applying the inclusion and exclusion criteria to each result reviewed through the Rayyan platform.

The studies included after this phase were searched and analyzed in full text, and a new screening process was carried out, justifying the inclusion and exclusion criteria. After this process, the eligible studies were included in the systematic review, and data extraction began. In disagreement, a third review author (JJB) was consulted.

Data extraction was conducted independently by two reviewers, and discrepancies were resolved by a third investigator. In cases where data were missing or incompletely reported in the included studies, we attempted to contact the corresponding authors to obtain additional information. If missing data could not be retrieved, we applied the following strategies. (1) For missing standard deviations in continuous outcomes, we estimated them from interquartile ranges or confidence intervals when available. (2) For dichotomous outcomes with missing event counts, we used sensitivity analyses with different plausible scenarios to assess the impact on pooled estimates. (3) Studies with excessive missing data (>20% of key outcomes) were excluded from quantitative synthesis. These approaches aimed to minimize bias and ensure robustness in the meta-analysis.

### 2.5. Risk of Bias Assessment

The risk of bias (RoB) was independently assessed using the RoB 2.0 tool. This tool evaluates five domains: (1) risk of bias arising from the randomization process, (2) deviations from intended interventions, (3) missing outcome data, (4) measurement of the outcome, and (5) selection of the reported result. Disagreements were resolved by discussion with a third author (JJB). RoB per domain and study was described as low, with some concerns, or high for RCTs.

### 2.6. Data Synthesis

Random-effects models with the inverse variance method were used for all meta-analyses comparing video laryngoscopy (VL) with direct laryngoscopy (DL). This approach was chosen due to the expected clinical and methodological heterogeneity across studies, particularly variations in patient populations, operator expertise, intubation settings (ICU, emergency department, operating room), and VL devices used. The Paule-Mandel method was employed to estimate the between-study τ^2^ variance, as it provides a robust approach when heterogeneity is present. Sensitivity analyses were conducted using fixed-effects models to assess the stability of the findings. The effects of video laryngoscopy (VL) compared to direct laryngoscopy on dichotomous outcomes were described with relative risks (RR) and their 95% confidence intervals (CI).

The continuity correction method adjusted for null events in one or two arms of the RCT. When more than five studies were found for meta-analysis, adjustments were made using the Hartung-Knapp method. Statistical heterogeneity of effects among RCTs was described by the I^2^ statistic, whose values represented low (<30%), moderate (30–60%), and high (>60%) levels of heterogeneity.

Sensitivity analyses were conducted to evaluate the robustness of findings. First, fixed-effects models were applied to assess the influence of small-study effects. Second, a leave-one-out analysis was performed by sequentially excluding each study to determine its impact on the overall effect size. Third, we conducted an additional sensitivity analysis excluding studies classified as having a high risk of bias to examine their influence on the pooled estimates. These approaches aimed to ensure the stability and reliability of our results. The metabin function of the R 3.5.1 meta library (https://www.r-project.org/; accessed on 15 December 2024) was used. Publication bias was assessed using funnel plots combined with Egger’s test for small-study effects. The funnel plots were visually inspected for asymmetry, which may indicate potential publication bias, and Egger’s regression test was used to provide a quantitative assessment. Funnel plots for primary outcomes are provided in Supplementary Figures S1 and S2.

### 2.7. GRADE Assessment

The GRADE methodology assessed the certainty of the evidence and the degree of recommendation regarding all outcomes. GRADE is based on domains such as risk of bias, inconsistency, indirectness, imprecision, and publication bias, which were evaluated. The outcomes determined the certainty of the evidence, which was described in the summary of results (SoF) tables created using the online software GRADEpro GDT (https://www.gradepro.org; accessed on 15 December 2024) (Supplementary Table S2).

This review informed the reference elements for systematic reviews and meta-analyses (PRISMA-2020, Supplementary Table S3). PROSPERO ID (CRD42024613946).

## 3. Results

### 3.1. Selection of Studies

**Figure 1:**
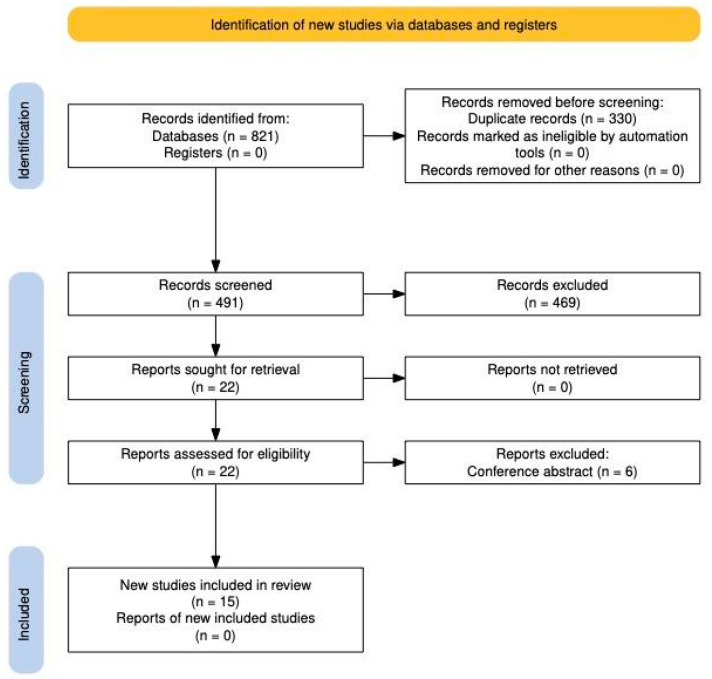
PRISMA 2020 Flow chart.

A total of 821 records were identified through database searches, with no records retrieved from registers. After removing 330 duplicates, 491 records were screened based on titles and abstracts, excluding 469 records that did not meet the inclusion criteria. Subsequently, 22 reports were assessed for eligibility through full-text review, and none were excluded due to retrieval issues. However, six reports were excluded as they were conference abstracts without sufficient data for inclusion. Finally, 15 studies met all eligibility criteria and were included in the final review, ensuring a rigorous selection process aligned with PRISMA guidelines [6,7,8,9,10,11,12,13,14,15,16,17,18,19,20] (Figure 1).

### 3.2. Characteristics of Included Studies

Table 1 describes the characteristics of the included studies. The studies were conducted in nations including China and the US and released between 2018 and 2023. Included were randomized, frequently unblinded studies. The studies’ aims varied, focusing on markers like overall procedural results, hemodynamic changes, and ease of intubation.

In most of the studies, no conflicts of interest were declared. More extensive multicenter trials involved up to 1420 patients, whereas more minor investigations involved 163 people. The inclusion criteria primarily focused on critically ill adult patients over 18 undergoing urgent or emergency intubation, frequently in the intensive care unit. Among the many exclusion criteria were patients under the age of 18, pregnant women, and those who were not suitable candidates for endotracheal intubation. The most common reasons for intubation were respiratory failure or airway protection, and the patients’ clinical conditions often included critical circumstances.

Table 2 demonstrates that the studies included a range of video laryngoscopy tools, including the McGrath and GlideScope, with interventions tailored to varying operator experiences and clinical contexts. All control group members used conventional direct laryngoscopy, primarily with the Macintosh blade. Procedure descriptions emphasized key elements such as preoxygenation methods, anesthetic protocols, and operator direction. Physician experience criteria differed across the investigations, ranging from highly experienced operators to general practitioners in intensive care units, highlighting disparities in the intubation domain. The trials examined success rates, operative duration, and hemodynamic stability outcomes.

### 3.3. Risk of Bias Assessment

**Figure 2:**
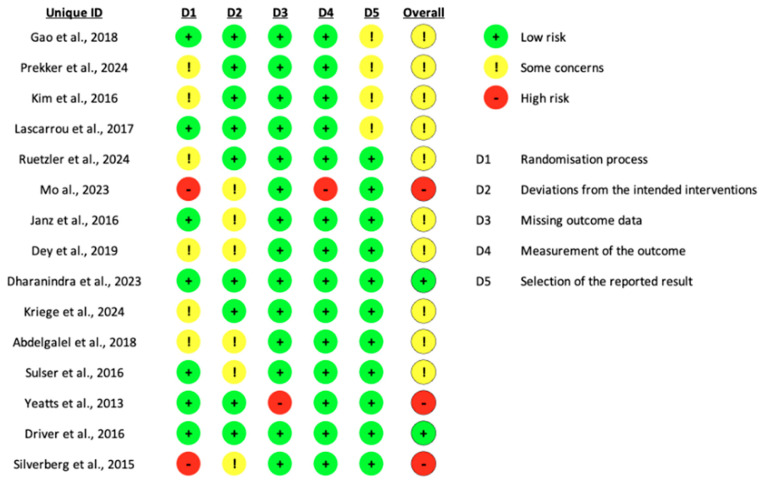
Risk of bias assessment [6,7,8,9,10,11,12,13,14,15,16,17,18,19,20].

Among the fifteen studies evaluated, three were at high risk of bias and ten had some concerns. Only two studies had a low risk of bias overall. Two studies were at high risk of bias in the randomization process, one in the deviation from the intended interventions and another study in the missing outcome data (Figure 2).

### 3.4. Effects of Video Laryngoscopy on Primary and Secondary Outcomes

**Figure 3:**
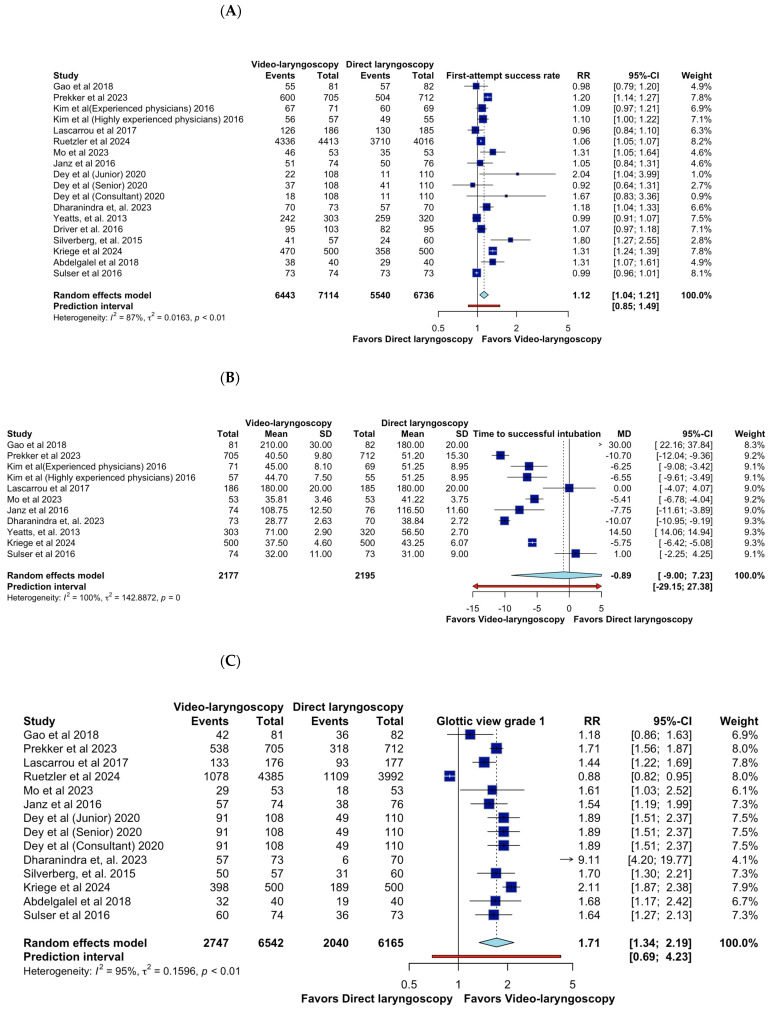

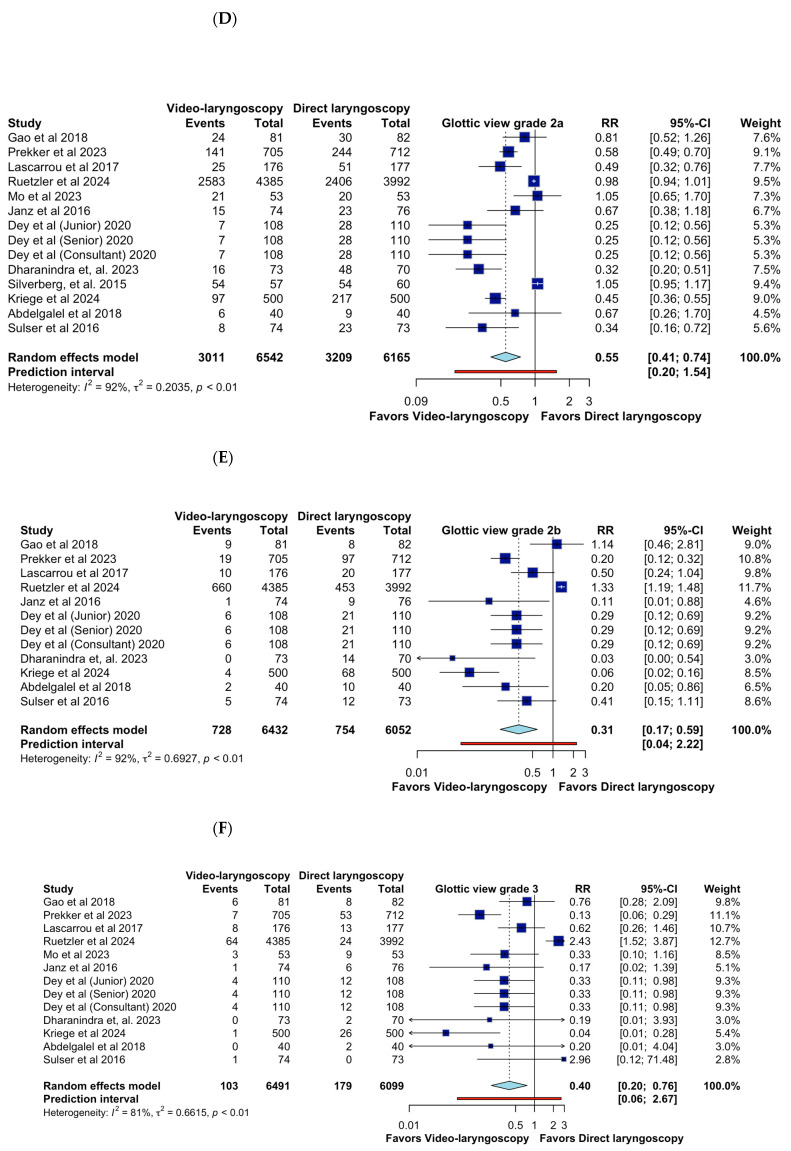
Effects of video laryngoscopy on primary outcomes. (**A**) First-attempt success rate; (**B**) Time to successful intubation; (**C**) Glottic view grade 1; (**D**) Glottic view grade 2a; (**E**) Glottic view grade 2b; (**F**) Glottic view grade 3 [6,7,8,9,10,11,12,13,14,15,16,17,18,19,20].

The meta-analysis of the first-attempt success rate of video laryngoscopy (VL) compared to direct laryngoscopy (DL) in critically ill adults undergoing tracheal intubation. The pooled estimate from a random-effects model reveals a relative risk (RR) of 1.12 (95% CI: 1.04–1.21, CoE Very Low), indicating a statisticallsy significant advantage of VL over DL in achieving successful intubation on the first attempt. However, substantial heterogeneity is evident, with an I^2^ of 87% and a τ^2^ of 0.0163, suggesting considerable variability across studies. The individual study estimates demonstrate a range of effect sizes, with some studies reporting a more pronounced benefit of VL, such as Silverberg et al. [20] (RR 1.80, 95% CI: 1.27–2.55) and Mo et al. [11] (RR 1.31, 95% CI: 1.05–1.64). The confidence intervals of several studies overlap with the line of no effect (RR = 1.0), emphasizing variability in individual study findings. This heterogeneity may stem from differences in study populations, operator experience, VL devices used, and clinical settings. For instance, Kim et al. [8] stratified their results based on physician experience, demonstrating that highly experienced practitioners achieved better outcomes with VL (RR 1.10, 95% CI: 1.00–1.22), whereas experienced physicians showed a more modest effect (RR 1.09, 95% CI: 0.97–1.21). The prediction interval (0.85–1.49) suggests that future studies could observe a range of outcomes, including those favoring DL, underscoring the uncertainty in generalizing these findings (Figure 3A).

Likewise, the forest plot displays the meta-analysis of time to successful intubation comparing video laryngoscopy (VL) and direct laryngoscopy (DL) in critically ill adults. The pooled mean difference (MD) of −0.89 s (95% CI: −9.00 to 7.23, CoE Very Low) suggests no statistically significant difference between techniques. However, substantial heterogeneity (I^2^ = 100%) indicates considerable variability among studies. Several studies report a shorter intubation time with VL, such as Prekker et al. [7] (MD −10.70, 95% CI: −12.04 to −9.36) and Dharanindra et al. [14] (MD −10.07, 95% CI: −10.95 to −9.19). Others, such as Yeatts et al. [18] (MD 14.50, 95% CI: 14.06 to 14.94), favor DL, contributing to the observed heterogeneity. The wide prediction interval (−29.15 to 27.38) underscores the uncertainty in future estimates, reinforcing that the impact of VL on intubation time is highly context-dependent. Operator experience, airway difficulty, and device type may influence outcomes, highlighting the need for further standardized research (Figure 3B).

Also, the set of forest plots evaluates the impact of video laryngoscopy (VL) versus direct laryngoscopy (DL) on glottic view quality, classified by Cormack–Lehane grades. For glottic view grade 1, the pooled relative risk (RR) is 1.71 (95% CI: 1.34–2.19; CoE Very Low), indicating that VL significantly improves optimal glottic visualization compared to DL. However, high heterogeneity (I^2^ = 95%) suggests variability in operator skill, patient characteristics, or device use. In grade 2a, VL demonstrates a protective effect (RR = 0.55, 95% CI: 0.41–0.74), favoring DL in achieving this level of glottic exposure. The substantial heterogeneity (I^2^ = 92%) underscores study differences in defining and achieving airway exposure. For grade 2b, VL significantly reduces the incidence (RR = 0.31, 95% CI: 0.17–0.59), reinforcing its advantage in preventing suboptimal visualization. Heterogeneity remains high (I^2^ = 92%), suggesting differences in study populations and procedural factors. Similarly, for grade 3, VL demonstrates a strong reduction in poor glottic views (RR = 0.40, 95% CI: 0.20–0.76), with heterogeneity (I^2^ = 81%) reflecting clinical variations. These findings confirm that VL provides superior glottic visualization, reducing the likelihood of difficult airway scenarios. However, high heterogeneity suggests the need for further research on standardizing VL use across clinical settings (Figure 3C–F).

**Figure 4:**
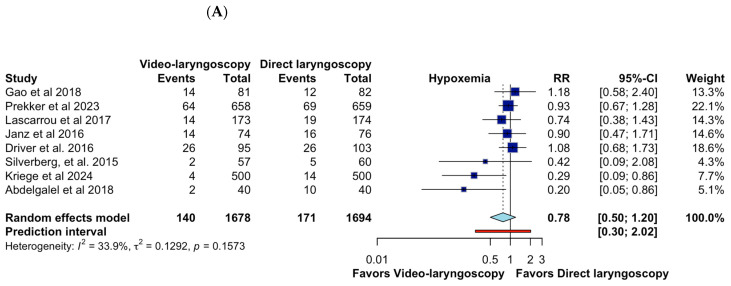

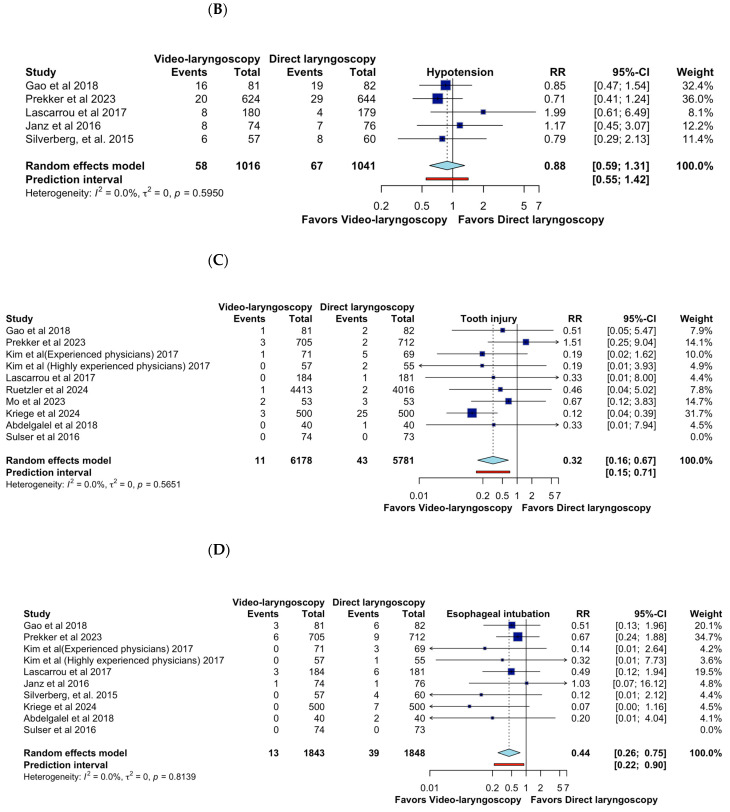

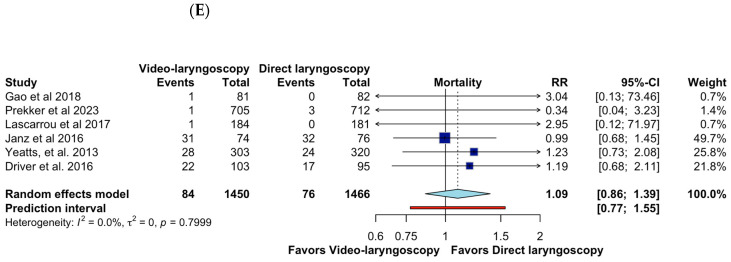
Effects of video laryngoscopy on secondary outcomes. (**A**) Hypoxemia; (**B**) Hypotension; (**C**) Tooth injury; (**D**) Esophageal intubation; (**E**) Mortality [6,7,8,9,10,11,12,16,17,19,20].

On the other hand, the forest plot evaluates the impact of video laryngoscopy (VL) versus direct laryngoscopy (DL) on the incidence of hypoxemia during tracheal intubation. The pooled relative risk (RR) of 0.78 (95% CI: 0.50–1.20; CoE Very Low) suggests a potential reduction in hypoxemia with VL; however, the confidence interval includes the null value, indicating no statistically significant difference. Heterogeneity is moderate (I^2^ = 33.9%), suggesting relatively consistent findings across studies. While some individual studies, such as Kriege et al. [15] (RR = 0.29, 95% CI: 0.09–0.86) and Abdelgalel et al. [16] (RR = 0.20, 95% CI: 0.05–0.86), favor VL, others show no significant effect. The prediction interval (0.30–2.02) indicates considerable uncertainty in future study outcomes, reinforcing the need for further research. Overall, while VL may reduce hypoxemia, the current evidence does not confirm a definitive advantage over DL (Figure 4A).

The forest plot assesses the impact of video laryngoscopy (VL) versus direct laryngoscopy (DL) on the incidence of hypotension following intubation. The pooled relative risk (RR) of 0.88 (95% CI: 0.59–1.31; CoE Very Low) suggests no statistically significant difference between VL and DL in terms of hypotension risk. Heterogeneity is negligible (I^2^ = 0%), indicating high consistency across studies. Individual study results vary, with some favoring VL (Prekker et al. [7], RR = 0.71, 95% CI: 0.41–1.24) and others showing no clear advantage. The prediction interval (0.55–1.42) suggests future studies may yield varying outcomes, but overall, VL does not appear to significantly influence hypotension rates compared to DL. Further research is needed to assess hemodynamic stability in different clinical contexts (Figure 4B).

Also, the forest plot evaluates the impact of video laryngoscopy (VL) versus direct laryngoscopy (DL) on the incidence of tooth injury during intubation. The pooled relative risk (RR) of 0.32 (95% CI: 0.16–0.67, CoE Very Low) indicates a statistically significant reduction in tooth injuries with VL. Heterogeneity is absent (I^2^ = 0%), suggesting highly consistent results across studies. The study by Kriege et al. [15] (RR = 0.12, 95% CI: 0.04–0.39) contributes substantially to the overall effect, reinforcing VL’s advantage in reducing dental trauma. The prediction interval (0.15–0.71) supports the robustness of these findings, suggesting that future studies are likely to confirm this protective effect. These results highlight VL’s benefit in minimizing airway-related trauma, particularly in challenging intubations (Figure 4C).

The forest plot evaluates the impact of video laryngoscopy (VL) versus direct laryngoscopy (DL) on the incidence of esophageal intubation. The pooled relative risk (RR) of 0.44 (95% CI: 0.26–0.75, CoE Very Low) indicates a statistically significant reduction in esophageal intubation with VL compared to DL. Heterogeneity is absent (I^2^ = 0%), demonstrating high consistency across studies. While some individual studies, such as Kriege et al. [15] (RR = 0.07, 95% CI: 0.00–1.16), show a pronounced benefit of VL, others report more modest effects. The prediction interval (0.22–0.90) reinforces the robustness of these findings, suggesting that future studies will likely confirm this protective effect. These results highlight VL’s advantage in reducing critical airway misplacement, making it a valuable tool in difficult airway management (Figure 4D).

Finally, the forest plot evaluates the impact of video laryngoscopy (VL) versus direct laryngoscopy (DL) on mortality. The pooled relative risk (RR) of 1.09 (95% CI: 0.86–1.39; CoE Very Low) suggests no statistically significant difference between VL and DL in terms of mortality rates. Heterogeneity is absent (I^2^ = 0%), indicating high consistency across studies. Individual study estimates vary widely, with some suggesting increased mortality risk with VL (Gao et al. [6], RR = 3.04, 95% CI: 0.13–73.46) and others favoring DL (Prekker et al. [7], RR = 0.34, 95% CI: 0.04–3.23), but all confidence intervals include the null effect. The prediction interval (0.77–1.55) reinforces the uncertainty in future outcomes, suggesting that mortality is likely influenced by multiple factors beyond the choice of laryngoscopy technique. Overall, these results indicate that while VL improves intubation success and reduces complications, its impact on patient survival remains inconclusive. Further research in high-risk populations is needed (Figure 4E).

### 3.5. Sensitivity Analysis

Sensitivity analyses confirmed the robustness of our findings. The application of fixed-effects models produced estimates consistent with those obtained using random-effects models, indicating that small-study effects did not substantially alter the results. The leave-one-out analysis, in which individual studies were sequentially removed, demonstrated that no single study disproportionately influenced the overall effect size. Furthermore, excluding studies classified as having a high risk of bias did not materially change the pooled estimates, suggesting that the observed effects were not driven by methodological weaknesses in certain studies. These findings reinforce the reliability of our conclusions regarding the comparative efficacy and safety of video laryngoscopy versus direct laryngoscopy in critically ill adults.

The certainty of evidence was assessed using GRADE. The first-attempt success rate showed a low certainty of evidence, primarily due to heterogeneity (I^2^ = 87%). The reduction in esophageal intubation with video laryngoscopy had a moderate certainty of evidence, while the mortality outcome was rated as very low certainty, indicating high uncertainty in its effect estimate (Table 3)

## 4. Discussion

This systematic review and meta-analysis evaluated the efficacy and safety of video laryngoscopy (VL) compared to direct laryngoscopy (DL) for tracheal intubation in critically ill adults. While the findings highlight certain advantages of VL, such as improved first-attempt success rates and reduced complications, the overall certainty of evidence remains low, limiting these results’ generalizability and clinical applicability.

The analysis demonstrated that VL improved the first-attempt success rate compared to DL, with a relative risk (RR) of 1.12 (95% CI: 1.04–1.21). Despite the statistical significance, the very low certainty of the evidence, influenced by high heterogeneity (I^2^ = 87%) and potential bias in the included studies, limits confidence in these findings. Time to successful intubation showed no significant difference between techniques (MD −0.89 s; 95% CI: −9.0 to 7.23), with substantial heterogeneity (I^2^ = 100%) that further reduces confidence in this outcome. These findings suggest that the clinical advantage of VL may depend on the context, including the operator’s experience and the clinical scenario.

These results align with previous findings that reported similar success rates in emergency settings [1], although our pooled analysis provides a more comprehensive assessment across multiple clinical contexts. The high heterogeneity observed likely reflects variations in operator experience, patient characteristics, and specific device configurations across studies. The authors observed that 80.8% of patients were successfully intubated on the first pass. However, in the GS group, 293 of the 313 (93.6%) were successful on the first pass (*p* < 0.001; risk difference of 0.128 with 95% CI of 0.0771–0.181). Similarly, a study showed that first-attempt success rates were 71.4% in the McGrath X-blade group versus 79.0% in the CMAC video laryngoscope group (*p* = 0.26), with an absolute difference of −7.6% (95% CI: −20%, 5.0%, *p*-value = 0.26) [21]. The authors concluded that in patients with in-line manual stabilization, without anticipated airway difficulty and in the hands of experienced operators, the McGrath X-blade provided superior glottic views but conferred no advantage over the C-MAC, with a longer median time to intubation compared to the CMAC video laryngoscope.

A key finding was the improved laryngeal associated with VL, as assessed by Cormack–Lehane grades. VL showed better performance for grades one and 2a, but the increased uncertainty in evidence counterbalanced this benefit. For grades 2b and 3, VL significantly reduced poor s compared to DL, suggesting a potential advantage in scenarios of difficult airway intubation. However, these results should be interpreted cautiously due to inconsistencies across studies. Consistent with our findings, previous studies concluded that Cormack–Lehane grades and glottic opening percentage scores were similar, and no significant differences were found between the two groups [6]. There was no statistical difference between the VL and DL groups regarding intubation complications (*p* = 0.310).

VL was associated with reduced complications, such as tooth injuries (RR 0.32; 95% CI: 0.16–0.67) and esophageal intubations (RR 0.44; 95% CI: 0.26–0.75), with low certainty of evidence. Other studies found no significant differences between complications such as dental injury or esophageal intubation. However, a study indicates that fewer esophageal intubations were observed in the video laryngoscopy cohort (0.4% vs. 1.3%, AOR = 0.2, 95% CI = 0.1 to 0.5) [22]. These findings are clinically relevant, as they highlight the potential safety benefits of VL. Conversely, outcomes such as hypoxaemia, hypotension, and mortality did not show significant differences between VL and DL, with very low certainty of evidence, underscoring the need for further research in these areas.

Currently, the lack of standardized definitions and inconsistent reporting of adverse events pose a challenge in accurately assessing the safety of video laryngoscopy (VL) across different patient populations and clinical settings. Previous studies have used varying criteria to define complications such as esophageal intubation, dental trauma, and hypoxemia, making direct comparisons difficult. To address this limitation, future research should adopt uniform definitions and consistent reporting protocols. The implementation of standardized guidelines will enable a more precise evaluation of VL-associated risks and facilitate cross-study comparisons, ultimately improving the clinical applicability of findings.

The effectiveness of VL is significantly influenced by operator skill and experience. While VL is designed to enhance glottic visualization and facilitate intubation, its success depends largely on clinician familiarity with the device and technical proficiency. Studies have shown that the learning curve for VL varies, and insufficient training may mitigate its potential benefits. Therefore, it is essential that training programs include specific VL modules and that periodic competency assessments are conducted to ensure effective implementation in clinical practice.

The integration of artificial intelligence (AI) into airway management represents a promising direction for future research. Recently, La via et al. (2024) [23] explored the use of AI models, including ChatGPT 4.0, in airway management, highlighting applications in education, clinical decision support, and patient communication. Furthermore, AI has the potential to improve difficult airway prediction by analyzing facial images and other clinical data, surpassing the limitations of traditional physical examinations. For instance, deep learning models have been developed to classify intubation difficulty based on facial image analysis, achieving 80.5% accuracy and 83.3% specificity. These tools could provide real-time assistance during intubation procedures, enhancing both safety and efficiency. However, clinical implementation requires rigorous validation and consideration of ethical and practical challenges, such as data privacy and the need for advanced technological infrastructure [23].

Our findings suggest that video laryngoscopy (VL) should be prioritized over direct laryngoscopy (DL) in scenarios involving difficult airways, given its higher first-attempt success and lower rates of esophageal intubation and dental trauma. This is particularly relevant in intensive care units (ICU) and emergency departments (ED), where rapid and successful airway management is critical. However, the benefit of VL may be operator-dependent, highlighting the need for standardized training programs to ensure proficiency. Additionally, while VL improves technical success, its lack of impact on mortality suggests that other factors, such as ventilation strategies and hemodynamic optimization, remain crucial for patient outcomes.

Several factors limit the robustness of our findings. First, most studies included in this meta-analysis were classified as having a high risk of bias or some concerns in critical domains, including randomization and outcome reporting. Second, the heterogeneity across studies was substantial, reflecting differences in study design, operator expertise, and patient populations. Finally, the very low certainty of evidence for many outcomes, as assessed using GRADE, reduces the confidence in the clinical applicability of these results.

These findings support the preferential use of video laryngoscopy in anticipated difficult airway scenarios and critically ill patients where maximizing first-pass success is essential. However, operator training and experience remain key determinants of success, emphasizing the need for structured airway management education. Future research should focus on optimizing training protocols and identifying specific clinical settings where VL provides the greatest advantage

Despite these limitations, the findings suggest that video laryngoscopy (VL) may offer advantages over direct laryngoscopy (DL), particularly in scenarios of predicted difficult airways. Its ability to enhance laryngeal and reduce complications, such as esophageal intubation and dental injury, is promising. However, the absence of significant improvements in critical outcomes, including mortality, underscores the need for cautious interpretation. These findings highlight that the incremental benefits of VL over DL may be context-dependent, particularly in emergencies managed by experienced providers.

Future research should address the methodological limitations identified in this meta-analysis to enhance the reliability and clinical applicability of the findings. High-quality, large-scale randomized controlled trials (RCTs) with standardized protocols are essential to reduce the substantial heterogeneity observed in primary outcomes and improve the overall certainty of the evidence. Such trials should prioritize rigorous methodologies, as most included studies demonstrated a high risk of bias or concerns in critical domains, particularly in randomization processes and outcome reporting.

Additionally, future studies should establish standardized definitions and consistent reporting of adverse events to evaluate better safety outcomes across diverse patient populations and clinical settings. Moreover, the role of operator expertise and experience in influencing the relative effectiveness of video laryngoscopy should be further explored.

## 5. Conclusions

Video laryngoscopy (VL) improves first-attempt success rates and reduces complications such as esophageal intubation and dental injury compared to direct laryngoscopy (DL), particularly in difficult airways. However, its universal adoption remains uncertain due to significant heterogeneity among studies and the low certainty of evidence for critical outcomes such as mortality. The decision to use VL should be individualized, considering patient-specific factors, operator expertise, and available resources. Future research should focus on standardizing training programs, evaluating device-specific performance, and assessing patient-centered outcomes to clarify its clinical impact.

## Figures and Tables

**Table 1:** Characteristics of included studies.

Author(s)	Year of Publication	Country	Type of Study	Study Objective	Financial Support	Conflicts of Interest	Total Sample Size	Inclusion Criteria	Exclusion Criteria	Clinical Status of the Patients
Gao et al. [6]	2018	China	Randomized, non-blinded trial comparing first-pass success rate of endotracheal intubation between VL (Med. Adult type Video Laryngoscope VL300M, Zhejiang UE Medical Corp., Xianju, China) and conventional DL.	To compare ease of intubation and hemodynamic changes with video laryngoscope (VL) (C-MAC) versus traditional laryngoscopy and to assess any complication such as arrhythmias, local injuries, bleeding, laryngospasm, regurgitation during intubation, and sore throat post-intubation.	Departmental and institutional support; video laryngoscopes and stylettes were provided by Verathon Inc.	None reported	163	ICU admission and need for endotracheal intubation to allow mechanical ventilation.	(1) Contraindications to endotracheal intubation (e.g., unstable spinal lesion); (2) age younger than 18 years; (3) currently pregnant or breast feeding.	The most common indication for intubation was acute respiratory failure.
Prekker et al. [7]	2023	USA	Multicenter, unblinded, randomized, parallel-group trial	To determine the effect of using a video laryngoscope as compared with a direct laryngoscope on the incidence of successful tracheal intubation on the first attempt in critically ill adults in the emergency department and ICU.	None reported	None reported	1420	Critically ill adults (age, ≥18 years) undergoing orotracheal intubation with the use of a laryngoscope. 1. Patient is located in a participating unit. 2. Planned procedure is orotracheal intubation using a laryngoscope. 3. Planned operator is a clinician expected to routinely perform tracheal intubation in the participating unit.	Patient is less than 18 years old. Pregnant. Prisoner. Immediate need for tracheal intubation precludes safe performance of study procedures. Operator has determined that use of a video laryngoscope or use of a direct laryngoscope is required or contraindicated for the optimal care of the patient.	Critically ill adults (age, ≥18 years) undergoing orotracheal intubation with the use of a laryngoscope were eligible.
Kim et al. (Experienced physicians and Highly experienced physicians) [8]	2016	Republic of Korea	Pospective randomized controlled study	To compare the success rate of ETI, speed of ETI, incidence of complications, and chest compression interruptions between experienced intubators using DL and VL in a clinical setting.	Konkuk University	None reported	140	IC was that an experienced intubator performed all ETIs during CPR for out-of-hospital or in-hospital cardiac arrest patients at the ED.	EC (1) ETIs per- formed on traumatic arrest patients wearing a cervical collar to protect a cervical injury, (2) ETIs performed by a physician who had <50 ETI experiences, and (3) ETIs with data loss or poor quality of recording.	Out-of-hospital or in-hospital cardiac arrest requiring ETI during CPR at the ED.
Lascarrou et al. [9]	2017	France	Non-blinded, multicenter, open-label, 2 parallel-group randomized clinical trial (RCT)	To test the hypothesis that routine use of the video laryngoscope for orotracheal intubation of patients in the ICU increased the frequency of successful first-pass intubation11 compared with use of the Macintosh direct laryngoscope.	Institutionally sponsored (Centre Hospitalier Département de la Vendée)	Some doctor reported receiving travel reimbursement, personal fees for serving on advisory boards and nonfinancial, consulting fees and grants. No other disclosures were reported.	371	ICU admission and need for orotracheal intubation to allow mechanical ventilation.	(1) Contraindications to orotracheal intubation (e.g., unstable spinal lesion), (2) insufficient time to include and randomize the patient (e.g., because of cardiac arrest), (3) age younger than 18 years, (4) currently pregnant or breastfeeding, (5) correctional facility inmate, (6) under guardianship, (7) without health insurance, (8) refusal by patient or next of kin, and (9) previous enrollment in an RCT with intubation as the primary end point (including previous inclusion in the present trial).	Simplified Acute Physiologic Score II, mean (SD). VL: 58.0 (21.0). VD:57.7 (21.8).
Ruetzler et al. [10]	2024	USA	Cluster-randomized and multiple cross-over clinical trial	To determine if the number of intubation attempts per surgical procedure is lower when using video laryngoscopy compared to direct laryngoscopy	Departmental and institutional support; the video laryngoscopes and stylets were provided by Verathon Inc.	None reported	8429	Adults aged 18 years or older scheduled for elective or emergent cardiac, thoracic, or vascular surgical procedures who required single-lumen endotracheal intubation for general anesthesia were enrolled.	Patients who had clinical indications for awake fiberoptic intubation, were already intubated, and those in whom clinicians refused to participate in this trial were excluded.	In total, 85% of the patients underwent elective surgical procedures; types of procedures: cardiac (75%), vascular (13%), thoracic (1.4%).
Mo et al. [11]	2023	China	Randomized clinical trial	To determine the safety and efficacy of video laryngoscopy compared to direct laryngoscopy.	None reported	None reported	106	Adult patients aged 18–60; endotracheal intubation in the emergency department due to critical illness; respiratory and cardiac arrests; signs of respiratory failure; hemodynamic instability and need for emergency tracheal intubation; and incomplete clinical data.	Respiratory malformations; pneumothorax; laryngeal edema; restricted mouth opening; and incomplete clinical data.	None reported
Janz et al. [12]	2016	USA	Prospective randomized comparative study	To determine if video laryngoscopy increases the first-attempt intubation rate	National Heart, Lung, and Blood Institute T32 Award: Vanderbilt Institute for Clinical and Translational Research Grante support	None reported	150	Not specifically reported: Age over 18, intended for the Intensive Care Unit for endotracheal intubation	None reported	Patients in both treatments with sepsis, septic shock, on vasopressors, cardiogenic shock, hemorrhagic shock, delirium, hepatic encephalopathy, myocardial infarction, drug overdose, and exacerbation of chronic obstructive pulmonary disease.
Dey et al. (Junior, Senior, and Consultant) [13]	2020	India	Prospective randomized comparative study	To determine if video laryngoscopy increases the first-attempt success rate of orotracheal intubation	None reported	None reported	228	Patients requiring elective endotracheal intubation in the Intensive Care Unit	Under 18 years old, pregnant and breastfeeding women, patients with facial trauma, cervical spine injury, lack of time for randomization and inclusion due to resuscitation attempts, unable to obtain informed consent.	None reported
Dharanindra et al. [14]	2023	India	Prospective randomized comparative study	To determine the effectiveness in glottic visualization, first-pass success, the time required for intubation, airway morbidities, and manipulations requiredof using King Vision video laryngoscope (KVVL) in intensive care unit (ICU) compared to Macintosh direct laryngoscope (DL).	None reported	None reported	143	All patients admitted to the ICU who required urgent and electiveendotracheal intubation	(a) Patients with upper airway deformities and (b) Patients with a known history of subglottic stenosis	Patients at ICU
Yeatts et al. [18]	2013	USA	Randomized controlled trial	To determinar si el uso de VL mejora la visualización durante la intubación y que resulta en un mejor manejo de la vía aérea y menor tiempo de intubación	Fondos internos del Programa de Trauma de la Escuela de Medicina de la Universidad de Maryland	None reported	623	All patients who required tracheal intubation in the trauma resuscitation unit (TRU): airway obstruction, hypo-ventilation, severe hypoxemia, cognitive impairment (Glasgow Coma Scale [GCS] score e 8), and hemorrhagic shock. Altered mental status, combativeness, and extreme pain	Patients with suspected laryngeal trauma or extensive maxillofacial injury who required an immediate surgical airway and patients with known or strongly suspected spinal cord injury for whom awake flexible fiber-optic intubation was indicated. The study also excluded patients in cardiac arrest on arrival as well as those who died in the TRU.	Airway obstruction, hypo-ventilation, severe hypoxemia, cognitive impairment (Glasgow Coma Scale [GCS] score e 8), and hemorrhagic shock. Altered mental status, combativeness, and extreme pain
Driver et al. [19]	2016	USA	Open-label, prospective, randomized, controlled trial	To compare first-pass success in patients undergoing emergency intubation with DL or VL using a C-MAC device.	None reported	The authors have disclosed that they do not have any potential conflicts of interest.	198	Adult patients who were to undergo emergency orotracheal intubation using DL were eligible for enrollment.	Patients were excluded if pregnant or a prisoner or if the treating physician planned an approach other than DL on the first intubation attempt.	None reported
Silverberg et al. [20]	2015	USA	Single-center prospective randomized controlled trial.		None reported	None	117	All patients who required urgent or emergent intubation in which the PCCM fellow was team leader either in the MICU or on the wards as part of the rapid response or code teams.	Patients were excluded if the intubation was elective for a procedure or had (1) a known history of difficult intubation, (2) presence of limited mouth opening, oropharyngeal masses, or swollen tongue, suggesting the inability to use a DL or GVL, or (3) oxygen saturation less than 92% after bag valve mask venti-lation.	Acute Physiology and Chronic Health Evaluation (APACHE) II scores reflected a significant degree of critical illness in both groups
Kriege et al. [15]	2024	Germany	Randomized controlled trial	We hypothesized that using the McGrathTM MAC (Medtronic, Minneapolis, MN, USA) videolaryngoscope for tracheal intubation during RSI in the operating theater would achieve a higher first pass tracheal success compared with standard direct laryngoscopy	None reported	None reported	1000	dult patients (aged ≥ 18 y) who were scheduled for elective or emergency surgery (within 6–24 h of a decision to operate) and for whom RSI was indicated	Patients with a known or anticipated difficult airway; patients with an airway difficulty score ≥ 9 [13]; pregnant or breastfeeding women; and patients with lifethreatening conditions requiring immediate surgery were not studied.	
Abdelgalel et al. [16]	2018	Egypt	Prospective randomized controlled study	We hypothesized that the use of a video laryngoscope would improve the success of the first attempt of endotracheal intubation witha better glottic view and reduce the incidence of complications.	Nil	None reported	120	Inclusion criteria were, age more than18 years, intensive care unit patients need emergency endotracheal intubation.	Exclusion criteria included patients required endotracheal intubation due to cardiac arrest, severe oxygen desaturation (Spo2 < 80%) and patients with diagnosed or predicted cervical spine injury.	
Sulser et al. [17]	2016	Switzerland	A randomized clinical trial	Specifically, we wanted to test the hypothesis that the C-MAC videolaryngoscope improves the first-attempt intubation success rate compared with direct laryngoscopy in patients undergoing emergency rapid sequence tracheal intubation (RSI) in the emergency room setting.	Financial support and sponsorship: only department and university funding was used.	None reported	147	Patients aged between 18 and 99 years undergoing emergency RSI in the emergency room	Owing to ethical considerations, patients suffering from major maxillofacial trauma, patients with an immobilized cervical spine, patients with an indication for awake fibreoptic guided intubation, and patients with ongoing cardiopulmonary resuscitation were not included.	

**Table 2:** Characteristics of interventions.

Author(s)	Year of Publication	Intervention Group (Video Laryngoscopy)	Control Group (Direct Laryngoscopy)	Description of the Procedure	Physician’s Experience	Statistical Method Use	Adjustment for Confounding Factors	Sensitivity Analysis
Gao et al. [6]	2018	VL (Med. Adult type Video Laryngoscope VL300M (UE Medical Corp., Zhejiang, China)	Conventional DL.	Preoxygenation was achieved using the device chosen by the bedside physician according to the standard ICU protocol, including a bag valve mask delivering oxygen at a flow of 10 L/min or greater for at least 3 min. Graded intravenous sedation without neuromuscular blocking agents was used to achieve optimal intubation conditions. The most commonly utilized sedative was propofol. Etomidate, midazolam, and fentanyl were used when propofol was unavailable or contraindicated.	All physicians working at ICU received hands- on training in the use of the video laryngoscope and conventional (direct) laryngoscope. And all the physicians involved had either worked at ICUs for at least 5 years or worked at ICUs for at least 1 year after receiving at least 2 months of anesthesiology training.	Continuous variables were expressed as the mean ± standard deviation (SD) or median with interquartile ranges (IQR), as appropriate. Comparisons of continuous variables between independent groups	No explicit additional adjustments are mentioned.	Conducted to evaluate effects of COVID-19, personnel refusals, and technical errors in randomization.
Prekker et al. [7]	2023	The operator was instructed to use a video laryngoscope on the first attempt at laryngoscopy. A video laryngoscope was defined as a laryngoscope with a camera and a video screen. The trial protocol did not specify the brand of video laryngoscope or the shape of the blade; both were selected by the operator. Operators were instructed to view the video screen while they performed laryngoscopy and inserted the endotracheal tube.	The operator was instructed to use a direct laryngoscope on the first attempt at laryngoscopy. A direct laryngoscope was defined as a laryngoscope without a camera or a video screen. The trial protocol did not specify the brand of direct laryngoscope or the blade shape (e.g., curved [Macintosh] or straight [Miller]).	All other aspects of the procedure were at the discretion of the treating clinicians, including the type of laryngoscope used on subsequent attempts. At all the trial sites, a stylet or bougie was routinely used during the first tracheal intubation attempt, and waveform capnography or colorimetric end-tidal carbon dioxide detection was used to confirm that the endotracheal tube was in the correct position.	In total, 91.5% of the intubations were performed by an emergency medicine resident or a critical care fellow. Operators had performed a median of 50 previous tracheal intubations (interquartile range, 25 to 92). The median proportion of previous intubations that operators had performed with the use		No explicit additional adjustments are mentioned.	Mixed-effects model: A generalized linear mixed-effects model was used to account for relevant covariates and site correlations, including age, sex, BMI, operator experience, and intubation location. Crossover analysis: The primary analysis was repeated, treating patients who switched laryngoscope types on the first attempt as not having a successful intubation on the first try. Independent observer data: The analysis was repeated using only cases with primary outcome data from an independent observer, excluding operator self-reported cases. Operator experience: The analysis was repeated among patients where the operator had comparable experience with both video and direct laryngoscopes (using a video laryngoscope in 25–75% of previous intubations).
Kim et al. (Experienced physicians and Highly experienced physicians) [8]	2016	ETI during CPR by the VLS user (GlideScope^®^.)	ETI during CPR by the DLs user	We randomized the intubator before arrival for out-of-hospital arrests and in-hospital arrests treated in the ED. Both intubator groups (DL users and VL users) were evenly allocated to the on-duty schedule before beginning their monthly duty. During the 2-year study period, all CPR performances at the ED were automatically recorded by a closed-circuit television system	Defined “experienced intubators” as those who had performed >50 successful ETIs. For Highly experienced intubators (>80 successful ETIs)	Logistic regression was used to compare the success rates of endotracheal intubation (ETI) between the direct laryngoscopy (DL) and video laryngoscopy (VL) groups. To compare the outcomes between two groups, a chi-square test or Mann–Whitney rank-sum test was used. To analyze the cumulative success rates associated with the time variables, including the censored data (oesophageal intubation or failed intubation), we used Kaplan–Meier analysis. Two-sided *p*-values < 0.05 were considered significant. Handling Missing Data: Multiple imputation was performed to address missing data, ensuring the robustness of the results. Multiple imputation was performed to address missing data, ensuring the robustness of the results.	The study does not explicitly mention adjustment for confounding factors beyond the consideration of operator experience in the logistic regression model.	reported an explicit sensitivity analysis
Lascarrou et al. [9]	2017	Intubation using a video laryngoscope (McGrath MAC)	Intubation using direct laryngoscopy (Macintosh)	All patients received general anesthesia. 1. Preoxygenation was achieved using the device chosen by the bedside physician according to the standard ICU protocol. 2. General anesthesia was then induced by injecting a hypnotic agent and a neuromuscular blocking agent. 3. Laryngoscopy was performed using the device allocated at random. 4. Intratracheal tube position was confirmed by analyzing the capnography curve over 4 breaths or more. 5. If the first-pass intubation attempt failed, the individual performing intubation chose between repeat laryngoscopy and an alternative intubation technique in accordance with French guidelines.	All physicians working at the participating ICUs received hands-on training in the use of the video laryngoscope and conventional (direct) laryngoscope. Specific equipment was provided to each participating center for the training sessions (e.g., size 3 and 4 blades of each laryngoscope type and mani- kins for intubation training). The first orotracheal intubation attempt was performed by nonexperts in 83.8% of patients and by experts in 16.2% of patients.	Mixed-effects logistic model to account for stratification factors. The model included center as a random effect and group and operator experience as fixed effects. The intention-to-treat principle was followed. A per-protocol analysis also was performed and excluded the patients who (1) did not meet inclusion or exclusion criteria, (2) did not receive invasive mechanical ventilation, or (3) had medical reasons for study withdrawal. Comparisons of the secondary outcomes were performed using the χ2 or Fisher exact test for qualitative data and the *t* test or the Wilcoxon rank sum test for quantitative data as appropriate. Intubation procedure duration was assessed using Kaplan–Meier curves and the log-rank test.	Adjustment for Operator Experience and Adjustment for Intubation Difficulty (MACOCHA Score):	A sensitivity analysis based on the MACOCHA score (which is made up of a Mallampati score of 3 or 4, apnea syndrome [obstructive], cervical spine limitation, opening mouth < 3 cm, coma, hypoxemia, and operator not being an anesthesiologist) for predicting difficult intubation was performed; when at least “This analysis helps to determine whether the study results are consistent even when accounting for the more challenging scenarios predicted by the MACOCHA score.” MACOCHA: scoring tool used to assess the difficulty of orotracheal intubation in patients, in emergency situations.
Ruetzler et al. [10]	2024	(n = 185). All patients received general anesthesia.	Similar-sized Macintosh laryngoscope, typically with stylets as per clinical preference	Supine position, general anesthesia induced with lidocaine, propofol or etomidate, fentanyl, and succinylcholine or rocuronium. Use of external maneuvers allowed to improve visualization.	Intubations performed by resident anesthesiologists, certified nurse anesthetists, and other trained professionals	The effect of video vs. direct laryngoscopy on the number of intubation attempts was assessed using a proportional odds cumulative logit generalized estimating equation model22 considering the outcome to be ordinal, and adjusting for con- founding by applying stabilized inverse probability of treatment weighting. Fixed effects for treatment, period (as a continuous number from 1 to number of periods), and operating room were included, with adjustment for within-patient correlation using an independence generalized estimating equation working correlation matrix. Multiple sensitivity analyses to assess the effects of COVID-19 precautions used by some clinicians, staff refusals to follow the randomization for various reasons, history of previous difficult intubation, and a technical error in the randomization during 3 weeks of the study were conducted The effect of video vs. direct laryngoscopy on intubation failure and on the collapsed composite of airway or dental injury were analyzed with a generalized linear mixed-effects log-binomial model (with log link to estimate relative risk for these binary outcomes) while adjusting for period (continuous), operating room and within-patient correlation, weighted by the stabilized weights. The effect of video laryngoscopy vs. direct laryngoscopy on maximum mean arterial pressure and heart rate in the 5 min after intubation was assessed using a linear mixed-effects model considering period within operating room as a random effect and adjusting for the within-patient correla- tion, weighted by the stabilized weights. A Wilcoxon-Mann–Whitney test was also conducted as sensitivity analysis. The treatment effect on the duration of intubation was assessed with a Wilcoxon–Mann–Whitney test.	Weighting by propensity scores with calibration	Conducted to assess the effects of COVID-19, staff refusals, and technical errors in randomization
Mo et al. [11]	2023	Video laryngoscope VDL (Model: vs. ~10 S)	Conventional laryngoscope	Supine position; if the patient did not cooperate or if complications arose during the procedure, a dose of midazolam was administered. The device was introduced into the mouth towards the right corner along the midline of the mouth and the epiglottis.	None reported	A paired-sample t value test was performed for intragroup distinction, and distinction between the 2 groups was tested using an independent-samples t value test. The count data, expressed as (n [%]), were tested with χ^2^/t.	None reported	None reported
Janz et al. [12]	2016	McGrath Video Laryngoscope, Glidescope Video Laryngoscope, or Olympus Video Bronchoscope	Curved Macintosh laryngoscope o hojas Miller	None reported	Intubations performed by an experienced operator	The data are expressed as median and interquartile range for continuous variables and frequencies for categorical variables. Between-group comparisons were conducted using the Wilcoxon’s rank-sum test for continuous variables and Fisher’s exact test for categorical variables. Logistic regression models were created to analyze the effect of VL on intubation on first laryngosocpy attempt while adjusting for (1) previous experience with the device at the time of the procedure and (2) previous experience with the device plus pre-specified baseline confounders.	“The operator’s experience with the device at the time of the procedure” and (2) “experience with the device with confounding baseline variables”	None reported
Dey et al. (Junior, Senior, and Consultant) ) [13]	2020	Karl Storz C-MAC Video Laryngoscope (Karl Storz GmbH & Co. KG, Tuttlingen, Germany)	Macintosh Laryngoscope	Preoxygenation was performed for at least 3 min using either a bag-mask ventilation with an oxygen flow of 15 L per minute or non-invasive ventilation with 100% oxygen. Induction medications included intravenous (IV) fentanyl (1–2 µg/kg) in all patients, with either propofol (1.5–2.0 mg/kg) IV or thiopental (3–5 mg/kg) IV. Neuromuscular blockade was achieved using succinylcholine (1–1.5 mg/kg) IV (except in patients with hyperkalemia or burns greater than 24 h) or rocuronium (0.9 mg/kg) IV. Laryngoscopies were performed using the randomly assigned method. After three failed intubation attempts, alternative techniques were used and were subsequently excluded from the analysis. A stylet was used when necessary, and external laryngeal manipulations were performed as indicated by the laryngoscopist. Alternative techniques such as the flexible bougie, laryngeal mask (LMA), intubating LMA, intubation endoscope, and cricothyrotomy kit were used based on the internal difficult airway algorithm. An intubation attempt was defined as the introduction of the laryngoscope and its subsequent removal with or without placement of the endotracheal tube (ETT). Successful placement of the ETT on the first attempt by an individual laryngoscopist was defined as first-attempt intubation success. Successful placement of the ETT was confirmed by auscultation and mainstream capnography (normal waveform for four or more respiratory cycles).	Anesthesiologists with a minimum of 50 video laryngoscopies were categorized into residents with more than 3 years, specialist physicians with 3–8 years, and consultants with more than 8 years based on their years of experience in anesthesia. Intubations were performed in the presence of two laryngoscopists, one of whom was either the specialist physician or the consultant anesthesiologist.	Baseline and demographic data were expressed as the mean ± standard deviation for Gaussian variables. The comparison of the two proportions was performed with the use of the chi-square test or Fischer’s exact test when appropriate. The comparisons of means and medians were performed using Student’s *t* test and Mann—Whitney test, respectively.	None reported	None reported
Dharanindra et al. [14]	2023	King Vision Video Laryngoscope with chaneled blade	Macintosh Direct Laryngoscope; the stylets might be used to facilitate endotracheal intubation.	All patients were pre-oxygenated using non-invasive ventilation (NIV) or bag-mask ventilation (BMV) and the drugs required for induction of anesthesia were decided upon the patient’s hemodynamic and clinical characteristics. Induction agents were used at the clinician’s discretion and included ketamine, propofol, etomidate, and fentanyl at recommended dosages. Rocuronium was used as a muscle relaxant at an appropriate dose. An arterial line was secured before induction when required. Rapid sequence intubation was performed in both groups.	None reported	Continuous variables were expressed in mean ± SD whereas, categorical variables were expressed in numbers and percentages (%). The continuous variables were analyzed using Student’s *t*-test and analysis of variance (ANOVA). The Chi-squared (χ^2^) test and Fisher’s exact test were used to compare the categorical variables.	None reported	None reported
Yeatts et al. [18]	2013	King Vision Video Laryngoscope	Macintosh Direct Laryngoscope	administration of 100% inspired oxygen for at least 1 min before intubation when possible, maneuvers to prevent passive regurgitation, the use of manual in-line stabilization in patients at risk for cervical spine injuries, and continuous monitoring of blood pressure, heart rate, oxygen saturation, and exhaled carbon dioxide.	Emergency medicine or anesthesiology residents with a minimum of 1 year of previous intubation experience performed the majority of the procedures under the direct supervision of an attending trauma anesthesiologist. The remaining intubations were performed by the attending anesthesiologist or a nurse anesthetist under attending guidance.	Student’s *t* test and W2 test were used to examine the null hypothesis, the results were expressed as mean (SD) and 95% confidence intervals (CIs). Wilcoxon rank-sum test was used to evaluate continuous data characterized by a nonnormal distribution. These results were expressed in medians and in- terquartile ranges (i.e., 25th through 75th percentiles). Among groups with different survival outcomes, multivariable logistic regression analysis was used to calculate adjusted odds ratio (AOR).	None reported	None reported
Driver et al. [19]	2016	C-MAC video laryngoscope with 3 or 4 blade	Direct laringoscopy	The first intubation attempt was performed with a C-MAC video laryngoscope with a size 3 or 4 Macintosh blade, as appropriate for the patient’s size. If the patient was assigned to the DL group, the C-MAC video screen was covered and the C-MAC blade was used as a direct laryngoscope. If the patient was assigned to the VL group, the C-MAC video screen was left uncovered and the treating physician was instructed to use indirect VL to perform endotracheal intubation. Because of the nature of the study, treating physicians were not blinded to the treatment assignment. If the first attempt failed, subsequent attempts could proceed with any device or technique.	Senior residents (postgraduate year 3 [PGY-3] or higher) perform the majority of tracheal intubations	The first-pass success rate was compared using the chi-square test. Secondary outcomes were compared using 95% confidence intervals (CIs).	None reported	None reported
Silverberg et al. [20]	2015	Videolaringoscope Glidescope	Direct laringoscopy Macintosh or Miller	All intubations were set up for both DL and GVL. Multiple-sized blades were available (Macintosh 3 and 4, Miller 4, and Glidescope 3 and 4). A rigid stylet was used routinely for all GVL intubations. Other devices, such as the bougie, bronchoscope, jet ventilation, and surgical airway equipment, were available if needed. When the operator was unsuccessful despite two attempts with any laryngoscope, they were required to switch devices or operators. a PCCM attending or an anesthesiologist must be present to supervise all intubations whenever possible.	First-year fellows performed 71% of the intubations.	Categorical variables are reported as counts and percentages. Baseline data were compared by *t* tests for continuous variables and by chi-square test or Fisher exact test for categorical variables. The time to intubation and number of intubations were compared using the two-sample Wilcoxon rank-sum (Mann–Whitney) test. Primary and secondary outcomes and complications were binary, and the chi-square test or Fisher exact test was used to compare outcomes and complications in the GVL and DL groups.	None reported	None reported
Kriege et al. [15]	2024	McGrath MAC videolaryngoscope (McGrath group)	Macintosh blade (direct laryngoscopy group)	Before induction of anesthesia, all patients received standard monitoring, which included electrocardiography, pulse oximetry and blood pressure (invasive or noninvasive). In the McGrath group, the tracheal tube was prepared with a stylet, while in the direct laryngoscopy group, it was prepared according to the local practice of each study center. No other airway adjuncts, such as a bougie, were permitted. Before starting, all patients were placed in a supine/reverse Trendelenburg (30° head up) position. After sufficient pre oxygenation, balanced anaesthesia was induced and maintained according to local standards and practices. After complete neuromuscular blockade was confirmed (train-of-four count of 0/4 or after muscular fasciculation stopped when using succinylcholine), intubation of the patient’s trachea was attempted.	Trainees < 6 years of anesthetic experience. Consultants ≥ 6 years of anaesthetic experience.	Binary data were analyzed using the v2 test or by Fisher’s exact test, if >20% of expected values were <5. Ordinal data were evaluated using the Wilcoxon-rank test. Kaplan–Meier curves and the log-rank test were used to compare comparative data. Continuous data were checked for normality by the Shapiro–Wilk W-test. Normal data were analysed by Student’s unpaired *t*-test and non-normal data were analysed by an independent sample Kruskal–Wallis test. Multiple logistic regression analysis using age, sex, ASA physical status, BMI, airway difficulty score and provider experience as potential explanatory variables for successful tracheal intubation within 120 s was assessed using Cox regression. We considered two tailed *p*-values < 0.05 to be significant. We used SPSS 9.4 (SAS Institute Inc., Cary, NC, USA) for statistical analysis.	None reported	None reported
Abdelgalel et al. [16]	2018	Glidescope Airtraq	Macintosh	After preoxygenation with 100% oxygen, rapid sequence induction (RSI) with cricoid pressure was performed. Induction agents included propofol 1–2 mg/kg or ketamine 1–2 mg/kg were titrated according to the patient response and hemodynamics. Fentanyl 1–2 mcg/kg and rocuronium 1 mg/kg were given then intubation was attempted after one minute.	Intubation was performed by ICU physician with more than 3 years of experience in anesthesia and intensive care and performed more than 30 intubations with each of Airtraq and Glidescope.	Continuous variables were expressed as mean ± SD and categorical variables were expressed as number (percentage). Continuous variables were checked for normality using Shapiro–Wilk test. One-way ANOVA test was used for comparing normally distributed data while Kruskal Wallis H test was used for non-normally distributed data. Percent of categorical variables were compared using Chi-square test. All tests were two sided. *p*-value < 0.05 was considered statistically significant. Statistical Package for Social Science version 20.0 (SPSS Inc., Chicago, IL, USA) and MedCalc for windows version 13 (MedCalc Software bvba, Ostend, Belgium) was used for analysis of all data.	None reported	None reported
Sulser et al. [17]	2016	C-MAC videolaryngoscopy with an appropriately sized Macintosh blade.	Direct laryngoscopy with an appropriately sized Macintosh blade.	Standard monitoring. Tracheal tubes were prepared with a hockey stick-shaped stilette. The backward, upward and rightward pressure maneuver was applied as indicated in all patients. Patients were placed in a supine position and tilted anti-Trendelenburg (about 308). Anesthesia for RSI was induced with fentanyl, propofol or thiopental, and succinylcholine or rocuronium, which ever was clinically appropriate. After complete muscle relaxation, confirmed by absence of palpable twitches in response to supramaximal train-of-four 1 Hz stimulation of the ulnar nerve at the wrist, the trachea was intubated as gently as possible.	Intubation was performed by one of three experienced anesthesia consultants.	The data are presented as mean SD or absolute numbers and percentage (%). Differences with regard to intubation success were reported with 95% Wilson confidence intervals (CI). Differences with regard to time to intubation were reported with 95% CI based on normal distribution. Binary data were compared using Fisher’s exact test and all other data were compared by the Wilcoxon–Mann–Whitney test. Exact two-tailed *p* values were calculated in SPSS for MAC (IBM SPSS Statistics, Version 22.0, Armonk, New York, NY, USA). *p* < 0.05 was considered significant.	None reported	None reported

**Table 3:** GRADE certainty of evidence for outcomes.

Outcome	Effect Size (RR or MD)	GRADE Certainty
First-attempt success	RR 1.12 (1.04–1.21)	Low
Time to intubation	MD −0.89 s (−9.0–7.23)	Very Low
Esophageal intubation	RR 0.44 (0.26–0.75)	Moderate
Hypoxemia	RR 0.78 (0.50–1.20)	Low
Mortality	RR 1.09 (0.86–1.39)	Very Low

## Data Availability

The data are contained within the article.

## Supplementary Materials

The following supporting information can be downloaded at https://www.mdpi.com/article/10.3390/jcm14061933/s1: Table S1. Strategy search; Table S2. GRADE assessment; Table S3. PRISMA checklist. Figure S1. Funnel plot for First-attempt success rate; Figure S2. Funnel plot for Time to successful intubation.
