# Supplementary material for: Video Versus Direct Laryngoscopy for Tracheal Intubation of Critically Ill Adults: A Systematic Review and Meta-Analysis
Source: J Clin Med. 2025 Mar 13;14(6):1933. doi: 10.3390/jcm14061933 (PMC11942891; doi:10.3390/jcm14061933)

Supplementary Materials

Supplementary Table S1. Search strategy

PUBMED	#1= (Adult OR Adults)<br><br>#2= ("Video Laryngoscopy" OR Videolaryngoscopy OR "Video Laryngoscopes")<br><br>#3= (Laryngoscopy OR "Laryngoscopy"[Mesh terms] OR "Direct Laryngoscopy" OR "Direct Laryngoscopes" OR Laryngoscopies OR "Laryngoscopic Surgical Procedures" OR "Laryngoscopic Surgical Procedure" OR "Procedure, Laryngoscopic Surgical" OR "Procedures, Laryngoscopic Surgical" OR "Surgical Procedure, Laryngoscopic" OR "Laryngoscopic Surgery" OR "Laryngoscopic Surgeries" OR "Surgeries, Laryngoscopic" OR "Surgery, Laryngoscopic" OR "Surgical Procedures, Laryngoscopic")<br><br>#4= ("Intubation, Intratracheal" OR "Endotracheal Intubation" OR "Endotracheal Intubations" OR "Intratracheal Intubation" OR "Intratracheal Intubations" OR "Intubation, Endotracheal")<br><br>#5= (“Randomized Controlled Trial” OR “Randomised Controlled Trial” OR “Clinical Trials, Randomized" OR “Trials, Randomized Clinical” OR “Controlled Clinical Trials, Randomized” OR “controlled clinical trial” OR “clinical trial”)	149
SCOPUS	#1= TITLE-ABS-KEY(Adult OR Adults)<br><br>#2= TITLE-ABS-KEY("Video Laryngoscopy" OR "Videolaryngoscopy" OR "Video Laryngoscopes")<br><br>#3= TITLE-ABS-KEY (Laryngoscopy OR "Laryngoscopy"[Mesh terms] OR "Direct Laryngoscopy" OR "Direct Laryngoscopes" OR Laryngoscopies OR "Laryngoscopic Surgical Procedures" OR "Laryngoscopic Surgical Procedure" OR "Procedure, Laryngoscopic Surgical" OR "Procedures, Laryngoscopic Surgical" OR "Surgical Procedure, Laryngoscopic" OR "Laryngoscopic Surgery" OR "Laryngoscopic Surgeries" OR "Surgeries, Laryngoscopic" OR "Surgery, Laryngoscopic" OR "Surgical Procedures, Laryngoscopic")<br><br>#4= TITLE-ABS-KEY ("Intubation, Intratracheal" OR "Endotracheal Intubation" OR "Endotracheal Intubations" OR "Intratracheal Intubation" OR "Intratracheal Intubations" OR "Intubation, Endotracheal")<br><br>#5= TITLE-ABS-KEY (“Randomized Controlled Trial” OR “Randomised Controlled Trial” OR “Clinical Trials, Randomized" OR “Trials, Randomized Clinical” OR “Controlled Clinical Trials, Randomized” OR “controlled clinical trial” OR “clinical trial”)	358

WEB OF SCIENCE	#1= (Adult OR Adults)<br><br>#2= ("Video Laryngoscopy" OR Videolaryngoscopy OR "Video Laryngoscopes")<br><br>#3= (Laryngoscopy OR "Laryngoscopy"[Mesh terms] OR "Direct Laryngoscopy" OR "Direct Laryngoscopes" OR Laryngoscopies OR "Laryngoscopic Surgical Procedures" OR "Laryngoscopic Surgical Procedure" OR "Procedure, Laryngoscopic Surgical" OR "Procedures, Laryngoscopic Surgical" OR "Surgical Procedure, Laryngoscopic" OR "Laryngoscopic Surgery" OR "Laryngoscopic Surgeries" OR "Surgeries, Laryngoscopic" OR "Surgery, Laryngoscopic" OR "Surgical Procedures, Laryngoscopic")<br><br>#4= ("Intubation, Intratracheal" OR "Endotracheal Intubation" OR "Endotracheal Intubations" OR "Intratracheal Intubation" OR "Intratracheal Intubations" OR "Intubation, Endotracheal")<br><br>#5= (“Randomized Controlled Trial” OR “Randomised Controlled Trial” OR “Clinical Trials, Randomized" OR “Trials, Randomized Clinical” OR “Controlled Clinical Trials, Randomized” OR “controlled clinical trial” OR “clinical trial”)	28
Embase	#1= 'adults'/exp OR adults<br>#2= 'videolaryngoscope'<br>#3= 'laryngoscopy'<br>#4= 'endotracheal intubation'<br>#5= 'randomized controlled trial'<br>#6= #1 AND #2 AND #3 AND #4 AND #5	286
	TOTAL OF RECORDS	821

Supplementary Table S2. GRADE assessment

Video Laryngoscopy compared to direct Laryngoscopy for tracheal intubation of critically ill adults

Patient or population: tracheal intubation of critically ill adults
Setting:
Intervention: Video Laryngoscopy
Comparison: direct Laryngoscopy

Outcomes	№ of participants (studies) Follow-up	Certainty of the evidence (GRADE)	Relative effect (95% CI)	Anticipated absolute effects	
				Risk with direct Laryngoscopy	Risk difference with Video Laryngoscopy
First-attempt success rate (FASR) assessed with: RR	13850 (18 RCTs)	⊕◯◯◯ Very low	**RR 1.12** (1.04 to 1.21)	822 per 1,000	**99 more per 1,000** (33 more to 173 more)
Time to successful intubation (TSI) assessed with: RR	4372 (11 RCTs)	⊕◯◯◯ Very low	-	The mean time to successful intubation was **76.46** seconds	MD **0.89 seconds lower** (9 lower to 7.23 higher)
Glottic view grade 2a (GG 2a) assessed with: RR	12707 (14 RCTs)	⊕◯◯◯ Very low	**RR 0.55** (0.41 to 0.74)	521 per 1,000	**234 fewer per 1,000** (307 fewer to 135 fewer)

Video Laryngoscopy compared to direct Laryngoscopy for tracheal intubation of critically ill adults

Patient or population: tracheal intubation of critically ill adults
Setting:
Intervention: Video Laryngoscopy
Comparison: direct Laryngoscopy

Outcomes	№ of participants (studies) Follow-up	Certainty of the evidence (GRADE)	Relative effect (95% CI)	Anticipated absolute effects	
				Risk with direct Laryngoscopy	Risk difference with Video Laryngoscopy
Glottic view grade 2b (GG 2b) assessed with: RR	12484 (12 RCTs)	⊕○○○ Very low	**RR 0.31** (0.17 to 0.59)	125 per 1,000	**86 fewer per 1,000** (103 fewer to 51 fewer)
Glottic view grade 3 (GG 3) assessed with: RR	12590 (13 RCTs)	⊕○○○ Very low	**RR 0.40** (0.20 to 0.76)	29 per 1,000	**18 fewer per 1,000** (23 fewer to 7 fewer)

***The risk in the intervention group** (and its 95% confidence interval) is based on the assumed risk in the comparison group and the **relative effect** of the intervention (and its 95% CI).

CI: confidence interval; **MD:** mean difference; **RR:** risk ratio

Video Laryngoscopy compared to direct Laryngoscopy for tracheal intubation of critically ill adults

Patient or population: tracheal intubation of critically ill adults
Setting:
Intervention: Video Laryngoscopy
Comparison: direct Laryngoscopy

Outcomes	№ of participants (studies) Follow-up	Certainty of the evidence (GRADE)	Relative effect (95% CI)	Anticipated absolute effects	
				Risk with direct Laryngoscopy	Risk difference with Video Laryngoscopy

GRADE Working Group grades of evidence
High certainty: we are very confident that the true effect lies close to that of the estimate of the effect.
Moderate certainty: we are moderately confident in the effect estimate: the true effect is likely to be close to the estimate of the effect, but there is a possibility that it is substantially different.
Low certainty: our confidence in the effect estimate is limited: the true effect may be substantially different from the estimate of the effect.
Very low certainty: we have very little confidence in the effect estimate: the true effect is likely to be substantially different from the estimate of effect.

Supplementary Table S3. PRISMA checklist

Topic	No.	Item	Location where item is reported
**TITLE**			
**Title**	1	Identify the report as a systematic review.	1
**ABSTRACT**			
**Abstract**	2	See the PRISMA 2020 for Abstracts checklist	
**INTRODUCTION**			
**Rationale**	3	Describe the rationale for the review in the context of existing knowledge.	2
**Objectives**	4	Provide an explicit statement of the objective(s) or question(s) the review addresses.	3
**METHODS**			
**Eligibility criteria**	5	Specify the inclusion and exclusion criteria for the review and how studies were grouped for the syntheses.	4
**Information sources**	6	Specify all databases, registers, websites, organisations, reference lists and other sources searched or consulted to identify studies. Specify the date when each source was last searched or consulted.	4
**Search strategy**	7	Present the full search strategies for all databases, registers and websites, including any filters and limits used.	4
**Selection process**	8	Specify the methods used to decide whether a study met the inclusion criteria of the review, including how many reviewers screened each record and each report retrieved, whether they worked independently, and if applicable, details of automation tools used in the process.	5
**Data collection process**	9	Specify the methods used to collect data from reports, including how many reviewers collected data from each report, whether they worked independently, any processes for obtaining or confirming data from study investigators, and if applicable, details of automation tools used in the process.	5

Topic	No.	Item	Location where item is reported
**Data items**	10a	List and define all outcomes for which data were sought. Specify whether all results that were compatible with each outcome domain in each study were sought (e.g. for all measures, time points, analyses), and if not, the methods used to decide which results to collect.	5
	10b	List and define all other variables for which data were sought (e.g. participant and intervention characteristics, funding sources). Describe any assumptions made about any missing or unclear information.	5
**Study risk of bias assessment**	11	Specify the methods used to assess risk of bias in the included studies, including details of the tool(s) used, how many reviewers assessed each study and whether they worked independently, and if applicable, details of automation tools used in the process.	5
**Effect measures**	12	Specify for each outcome the effect measure(s) (e.g. risk ratio, mean difference) used in the synthesis or presentation of results.	5
**Synthesis methods**	13a	Describe the processes used to decide which studies were eligible for each synthesis (e.g. tabulating the study intervention characteristics and comparing against the planned groups for each synthesis (item 5)).	5
	13b	Describe any methods required to prepare the data for presentation or synthesis, such as handling of missing summary statistics, or data conversions.	5
	13c	Describe any methods used to tabulate or visually display results of individual studies and syntheses.	5
	13d	Describe any methods used to synthesize results and provide a rationale for the choice(s). If meta-analysis was performed, describe the model(s), method(s) to identify the presence and extent of statistical heterogeneity, and software package(s) used.	5
	13e	Describe any methods used to explore possible causes of heterogeneity among study results (e.g. subgroup analysis, meta-regression).	5
	13f	Describe any sensitivity analyses conducted to assess robustness of the synthesized results.	5
**Reporting bias assessment**	14	Describe any methods used to assess risk of bias due to missing results in a synthesis (arising from reporting biases).	5

Topic	No.	Item	Location where item is reported
**Certainty assessment**	15	Describe any methods used to assess certainty (or confidence) in the body of evidence for an outcome.	6
**RESULTS**			
**Study selection**	16a	Describe the results of the search and selection process, from the number of records identified in the search to the number of studies included in the review, ideally using a flow diagram.	7
	16b	Cite studies that might appear to meet the inclusion criteria, but which were excluded, and explain why they were excluded.	7
**Study characteristics**	17	Cite each included study and present its characteristics.	7
**Risk of bias in studies**	18	Present assessments of risk of bias for each included study.	8
**Results of individual studies**	19	For all outcomes, present, for each study: (a) summary statistics for each group (where appropriate) and (b) an effect estimate and its precision (e.g. confidence/credible interval), ideally using structured tables or plots.	8
**Results of syntheses**	20a	For each synthesis, briefly summarise the characteristics and risk of bias among contributing studies.	8
	20b	Present results of all statistical syntheses conducted. If meta-analysis was done, present for each the summary estimate and its precision (e.g. confidence/credible interval) and measures of statistical heterogeneity. If comparing groups, describe the direction of the effect.	8
	20c	Present results of all investigations of possible causes of heterogeneity among study results.	8
	20d	Present results of all sensitivity analyses conducted to assess the robustness of the synthesized results.	8
**Reporting biases**	21	Present assessments of risk of bias due to missing results (arising from reporting biases) for each synthesis assessed.	8
**Certainty of evidence**	22	Present assessments of certainty (or confidence) in the body of evidence for each outcome assessed.	8
**DISCUSSION**			
**Discussion**	23a	Provide a general interpretation of the results in the context of other evidence.	9

Topic	No.	Item	Location where item is reported
	23b	Discuss any limitations of the evidence included in the review.	10
	23c	Discuss any limitations of the review processes used.	11
	23d	Discuss implications of the results for practice, policy, and future research.	11
**OTHER INFORMATION**			
**Registration and protocol**	24a	Provide registration information for the review, including register name and registration number, or state that the review was not registered.	6
	24b	Indicate where the review protocol can be accessed, or state that a protocol was not prepared.	6
	24c	Describe and explain any amendments to information provided at registration or in the protocol.	NA
**Support**	25	Describe sources of financial or non-financial support for the review, and the role of the funders or sponsors in the review.	NA
**Competing interests**	26	Declare any competing interests of review authors.	NA
**Availability of data, code and other materials**	27	Report which of the following are publicly available and where they can be found: template data collection forms; data extracted from included studies; data used for all analyses; analytic code; any other materials used in the review.	NA

Supplementary Figure S1. Funnel plot for First-attempt success rate

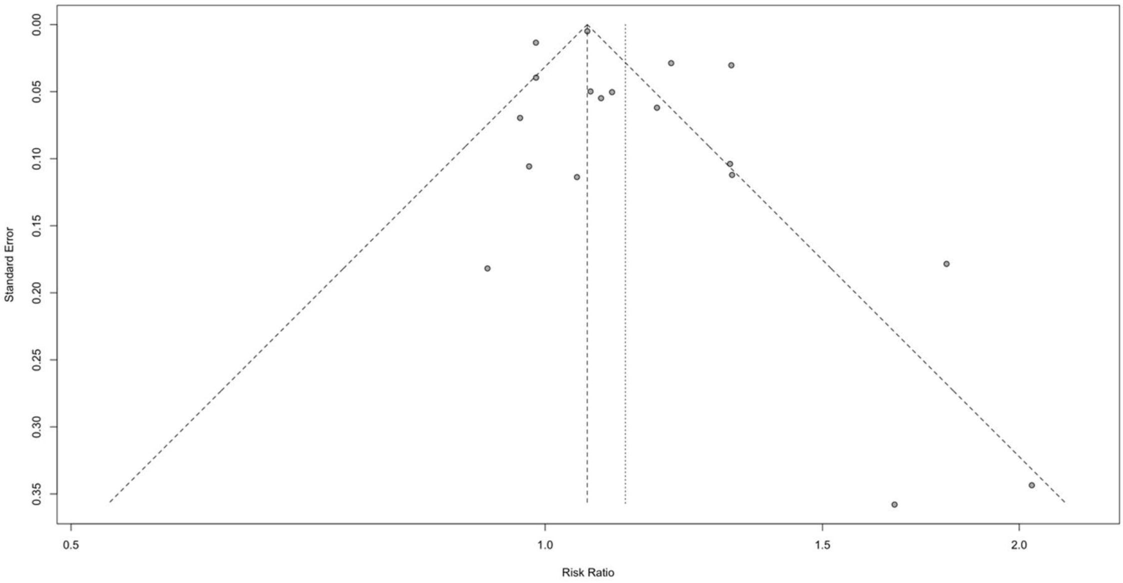

Supplementary Figure S2. Funnel plot for Time to successful intubation

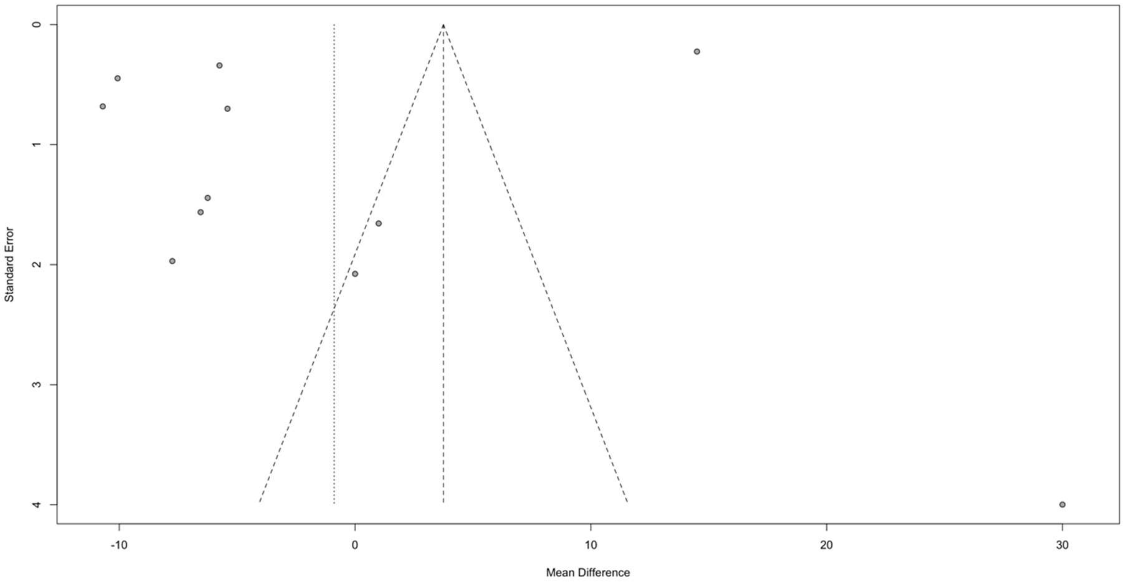

Supplement: Supplementary file 1 [file jcm-14-01933-s001.zip › jcm-3505216-supplementary.pdf]
